# Post-traumatic diaphragmatic rupture with pericardial denudation: A case report

**DOI:** 10.1016/j.ijscr.2021.105970

**Published:** 2021-05-15

**Authors:** A. El Bakouri, A. El Karouachi, M. Bouali, K. El Hattabi, F.Z. Bensardi, A. Fadil

**Affiliations:** a Visceral Surgery Emergency Department 35, University hospital center Ibn Rochd, Casablanca, Morocco; b Faculty of Medicine and Pharmacy, Hassan 2 university, Casablanca, Morocco

**Keywords:** Diaphragmatic rupture, Abdominal trauma, Treatment, Pericardio-diaphragmatic rupture, Case report

## Abstract

**Introduction:**

Post-traumatic diaphragmatic rupture is a lesion of variable severity. It is a rare and difficult to diagnose pathology, it has been found in 0.4% of all traumatized patients and in 1.9% of blunt traumas. It can be associated with abdominal andthoracic lesions, particularly cardiac, which can be life-threatening.

**Materials and methods:**

Our work is a retrospective case report with a descriptive aim concerning a patient operated for a post-traumatic diaphragmatic rupture within the department of general surgery of CHU Ibn Rochd Casablanca. This work has been reported in line with the SCARE 2020 criteria (17).

**Case presentation:**

A 60-year-old patient was admitted to the visceral surgical emergency department following a work accident (crushing between two carts) causing a thoraco-abdominal impact point trauma without initial loss of consciousness, nor externalized digestive hemorrhage or associated signs, but with a general condition alteration. The patient was conscious, dyspneic with a blood pressure of 100/50 mmHg and afebrile. Physical examination showed diffuse abdominal sensibility. The thoraco-abdomino-pelvic CT scan revealed the presence of a left thoracic hernia with gastric, colic and epiploic contents through a lateral defect of the left diaphragmatic dome. The decision was to directly send the patient to the operating room. Exploration found a large left diaphragmatic breach of 20 cm, a denudation of the pericardia, a medium-abundant hemoperitoneum and a hematoma of the right mesocolon. The procedure consisted of right hemicolectomy with ileocolic anastomosis, treatment of a diaphragmatic breach with a 2-silk raphia, thoracic drainage with a Joly drain, pericardial drainage with a Joly drain, pre-anastomotic drainage with 2 delbet slides, drainage of the Douglas and left subthreshold with 2 Salem catheters. The post-operative follow-up was simple.

**Discussion:**

Diaphragmatic rupture is a rare and difficult to diagnose condition. Traumatic diaphragmatic rupture (TDR) was found in 0.4% of all traumatized patients and in 1.9% of blunt trauma. Associated lesions of the spleen, liver and/or lungs were found in more than 30% of cases, with an overall mortality rate of 26.8% (1). Pericardial rupture following blunt chest trauma is rare and associated with a high mortality rate ranging from 30% to 64% (9).

The physiopathology of this type of injury is not well understood, but the most accepted hypothesis describes an increase in intra-abdominal pressure due to a blunt creating a sufficiently high-pressure gradient between the chest and the abdomen to cause a diaphragmatic rupture. The common clinical symptoms of a diaphragmatic rupture are a marked respiratory distress and diffuse abdominal pain but it can be asymptomatic. Medical imaging exams visualize the ascended organs but it's more difficult to visualize the rupture itself. The chest X-ray is currently the first examination to be requested (4) and also helps in the diagnosis of injuries and diaphragm rupture (13). Surgical treatment includes the reduction of any visceral hernia, repair of the diaphragm and restoration of circulation, breathing and digestive functions. Laparotomy is generally used because of the complete exploration of the abdominal viscera, although it is easier to reduce herniated tissue and repair the diaphragm.

**Conclusion:**

Diaphragmatic rupture with denudation of the heart is rare with poor prognosis and requires emergency surgery with close postoperative monitoring in the intensive care setting.

**Summary:**

Post-traumatic diaphragmatic rupture is a lesion of variable severity. It is a rare and difficult to diagnose pathology, it has been found in 0.4% of all traumatized patients and in 1.9% of blunt traumas. The lesions are more frequent in the left diaphragmatic dome compared to the right one, and exceptionally bilateral. Pericardial rupture following blunt chest trauma is rare and associated to a high mortality rate.

It is often unrecognized and goes unnoticed in the acute phase. The most common clinical symptoms of diaphragmatic ruptures are respiratory distress and diffuse abdominal pain, as it can be asymptomatic. Its diagnosis is essentially radiological using CT scan, and requires emergency surgical treatment as soon as the diagnosis is suspected, in order to avoid the dreaded complications.

Traumatic diaphragmatic rupture remains a diagnostic and therapeutic challenge.

We report the case of a patient who presented a post-traumatic diaphragmatic rupture with pericardial damage operated in the visceral emergency department at the Ibn Rochd Hospital c in Casablanca, Morocco.

## Highlights

• Pericardial rupture following blunt chest trauma is associated with a high mortality rate
• It requires emergency surgical treatment
• Intra-abdominal pressure due to a blunt creating a sufficiently high-pressure gradient to cause a diaphragmatic rupture

## 1 Introduction

Diaphragmatic ruptures are rare and difficult to diagnose. It occurs in less than 0.5% of all trauma cases, the majority being caused by a penetration mechanism (67%), followed by blunt mechanism injuries (33%) [1]. Diaphragmatic lesions on the left side are more frequently reported in the literature. Bilateral injuries are extremely rare [2]. The physiopathology of this type of injury is not well understood [1]. Traumatic diaphragmatic ruptures remain a diagnostic and therapeutic challenge for the emergency physician and surgeon. They can be associated with abdominal thoracic lesions, particularly cardiac, which can be life-threatening [3]. This work has been reported in line with the SCARE 2020 criteria [17].

## 2 Observation

A 60-year-old patient, known to be a chronic 20 pack-year smoker, is admitted to the visceral surgical emergency department following a work accident (crushing between two carts) causing a thoraco-abdominal impact point trauma without initial loss of consciousness, nor externalized digestive hemorrhage or associated signs, but with a general condition alteration.

The patient was conscious, dyspneic with a blood pressure of 100/50 mmHg and afebrile. Physical examination showed diffuse abdominal sensibility.

Concerning the paraclinical evaluation, the thoraco-abdomino-pelvic CT scan (A) revealed the presence of a left thoracic hernia with gastric, colic and epiploic contents through a lateral defect of the left diaphragmatic dome which measures 4 cm, an effusion on each side of the hernia with collapse of the adjacent lung and a peri-hepatic effusion at the level of the right iliac fossa, its density is measured at 50 HU, probably related to a hemoperitoneum (Fig. 1).

**Image 1:**
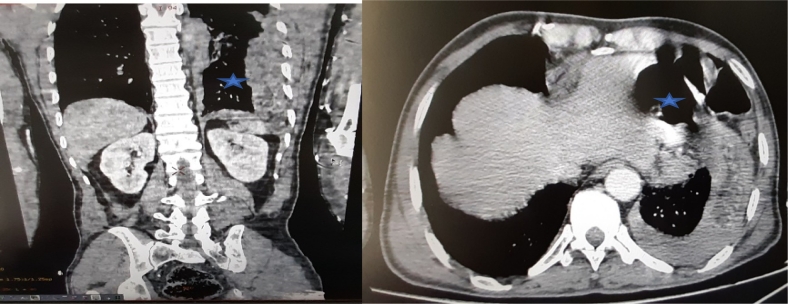
Thoraco-abdomino-pelvic scan shows an ascent of abdominal contents at the thoracic level through a defect of the diaphragmatic dome .

The decision was to directly send the patient to the operating room.

During exploration, a large left diaphragmatic breach of 20 cm, a denudation of the pericarde, medium-abundant hemoperitoneum and a hematoma of the right mesocolon with devascularization and pre-perforative lesions of the ascending colon were found (Fig. 2). The procedure consisted of right hemicolectomy with ileocolic anastomosis, treatment of a diaphragmatic breach with a 2-silk raphia, thoracic drainage with a Joly drain, pericardial drainage with a Joly drain, pre-anastomotic drainage with 2 delbet slides, drainage of the Douglas and left subthreshold with 2 Salem catheters.

**Image 2:**
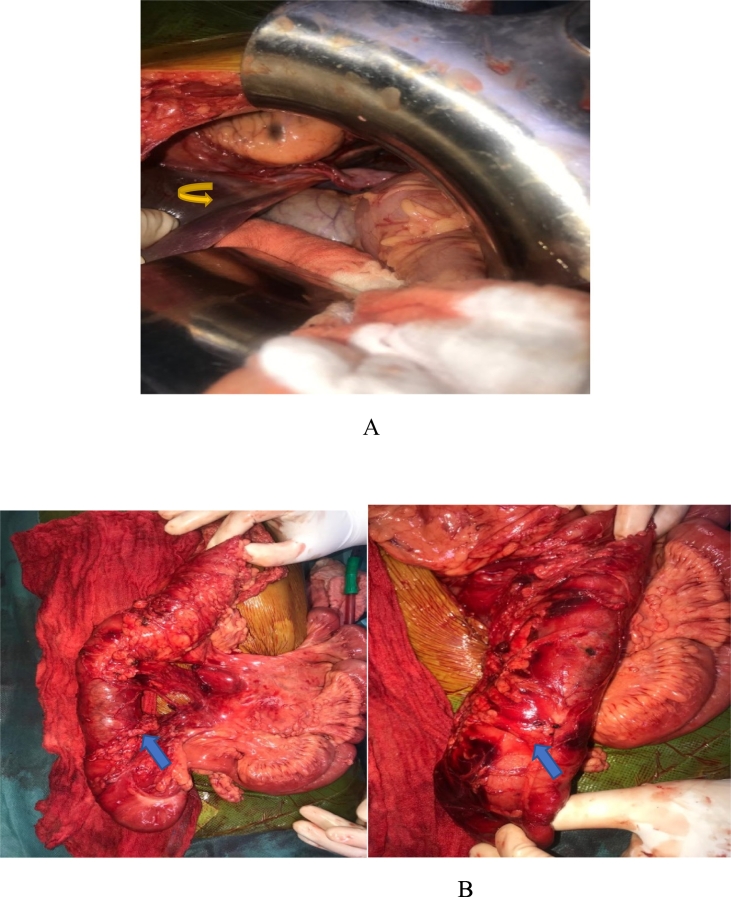
Images of the surgical exploration showing pericardic denudation  after diaphragmatic post traumatic rupture  (A) with devascularization and pre-perforative lesions of the ascending colon  (B).

The post-operative follow-up was simple. The patient was able to quit the service at D12 after removal of all drains and blades. Monitoring was very favorable over a 3-month follow-up.

## 3 Discussion

Diaphragmatic rupture, as a rare and difficult to diagnose condition, was described for the first time by Sennertus in 1541 [1].

Traumatic diaphragmatic rupture (TDR) was found in 0.4% of all traumatized patients and in 1.9% of blunt trauma. 80% of traumatic diaphragmatic ruptures were found on the left side and 20% on the right side. It was bilateral in about 3% of the cases. The incidence of post-traumatic diaphragmatic rupture among cases of penetrating traumas is 20 to 59% with gunshots and 15 to 32% with stab wounds [[4], [5], [6]].

60 to 70% of traumatized victims with diaphragmatic rupture in car accidents are men, 30 to 40% are women, with an age going from 30 and 45 years old. A fall from altitude can also rupture the diaphragm [7].

The right diaphragm is congenitally stronger than the left and is partially protected by the liver, which can distribute the pressure over a larger area [8].

Associated lesions of the spleen, liver and/or lungs were found in more than 30% of cases, with an overall mortality rate of 26.8% [1].

Pericardial rupture following blunt chest trauma is rare and associated with a high mortality rate ranging from 30% to 64% [9].

Patients with blunt injuries tend to be older, with a median age of 44 years. In contrast, those with penetrating injuries have a median age of 31 years. Mortality is highest in patients with acute blunt injury associated to other conditions. Mortality due to late consultation with herniation of the abdominal contents in the thorax, following prior penetrating trauma, is about 20% and is significantly higher in cases of intestinal strangulation [10].

The physiopathology of this type of injury is not well understood, but the most accepted hypothesis describes an increase in intra-abdominal pressure due to a blunt creating a sufficiently high-pressure gradient between the chest and the abdomen to cause a diaphragmatic rupture. Normally there is a positive gradient of 7 to 20 cmH2O between intraperitoneal and intrapleural pressures, but in blunt injuries this positive pressure gradient can exceed 100 cmH2O, resulting in a rupture [1].

This is the case of a patient who is admitted for a post-traumatic rupture of the left diaphragmatic dome with denudation of the heart.

The etiology of diaphragmatic trauma can be either a slowdown in car accident, falls from altitude and crush injuries to the lower chest or upper abdomen, which are the most common causes of blunt diaphragm injuries [4].

Traumatic rupture of the diaphragm may be asymptomatic [7].

The common clinical symptoms of a diaphragmatic rupture are a marked respiratory distress and diffuse abdominal pain [7,11].

This is the case of our patient who presented with a diffuse abdominal sensibility on clinical examination with a respiratory distress.

Some patients may present with chest and/or abdominal pain depending on the initial point of impact and may be asymptomatic for some [12].

Traumatic diaphragmatic rupture can be divided into three phases: acute, latent and obstructive. During the acute phase: abdominal pain, chest pain and cardiorespiratory dysfunction are usually present. During the latent phase, clinical symptoms of the hernia will be found, such as gastrointestinal disorders, pain in the upper left quadrant or chest, dyspnea/orthopnea, decreased breath sounds. In the obstructive phase, the above mentioned symptoms progress and signs of peritonitis may develop [4].

On further examination, medical imaging exams visualize the ascended organs but it's more difficult to visualize the rupture itself [7].

The chest X-ray is currently the first examination to be requested [4] and also helps in the diagnosis of injuries and diaphragm rupture [13].

In our case, the chest X-ray was not performed.

Computed tomography (CT) is the imaging technique of second choice, although the axially oriented diaphragm is not always well demonstrated on conventional CT. In a single-layer spiral CT study, the sensitivity for left and right-sided diaphragmatic lesions was 78% and 50%, but with multi-detector CT (MDCT) specificity was 100% and 83% for left and right-sided lesions. Currently, in cases of polytrauma and trauma, including patients with thoraco-abdominal trauma, a whole body CT scan is performed [4].

The CT scan is more sensitive and can confirm the diagnosis in 68.7% of diaphragmatic ruptures [14].

Abdominal ultrasound and chest X-ray are the examinations performed in principle at the emergency service for an unstable patient. For a stable or stabilized patient, the full-body CT scan injected at the arterial and portal times is the reference imaging technique for the lesion assessment [6].

MRI has also been described as a useful imaging adjunct, but because it is generally a time demanding modality, it may be less useful in acute trauma situations [12].

Diagnostic laparoscopy remains an excellent tool for the detection of hemoperitoneum, solid organ damage and diaphragmatic lacerations; And Thoracoscopy has a diagnostic accuracy of 98–100% for diaphragmatic injuries. The diagnosis may even be unsuspected and can only be made by laparotomy in 50% of cases of hemorrhage [15].

A recognized diaphragmatic rupture constitutes an operative indication, as soon as the diagnosis is made [16].

Surgical treatment includes the reduction of any visceral hernia, repair of the diaphragm and restoration of circulation, breathing and digestive functions. Surgical approaches, such as thoracotomy, laparotomy, and thoracotomy with laparotomy, are often used to treat a diaphragmatic rupture. Laparotomy is generally used because of the complete exploration of the abdominal viscera, although it is easier to reduce herniated tissue and repair the diaphragm [16].

Thoracotomy may be necessary for patients with chronic injuries, to safely separate adhesions between the abdominal organs and the pleura [8].

The actual repair is simple: once the contents of the hernia have been reduced, the rupture of the diaphragm can be closed with non-absorbable sutures. A chest drain should be left in the chest for a few days [10].

## 4 Conclusion

Traumatic diaphragmatic rupture remains a diagnostic challenge. CT scan with diaphragm reconstruction is useful for both diagnosis and differential diagnosis. Surgical therapy after diagnosis is the best treatment. Diaphragmatic rupture with denudation of the heart is rare with poor prognosis and requires emergency surgery with close postoperative monitoring in the intensive care setting.

## Funding

There are no sources of funding.

## Ethical approval

It is a case report and not a study.

## Consent

Written informed consent was obtained from the patient for publication of this case report and accompanying images. A copy of the written consent is available for review by the Editor-in-Chief of this journal on request.

## Registration of research studies

researchregistry2464.

## CRediT authorship contribution statement

This work was carried out in collaboration among all authors. All authors contributed to the conduct of this work. They also declare that they have read and approved the final version of the manuscript.

## Declaration of competing interest

The authors report no declarations of interest.
